# American Dental Convention

**Published:** 1861-11

**Authors:** 


### AMERICAN DENTAL CONVENTION.

### SEVENTH ANNUAL MEETING.

THOUGH headed as above, it is no part of the plan of this article to notice the Convention, as such, for we were not there; nor to review its proceedings, for we can not ascertain fully what they were. But there are, in some of the Journals, reports of the sayings and doings of the late meeting; and, for reasons *satisfactory to ourselves*, we propose to say a little in regard to these sayings and doings, and will take the report of the *Cosmos* as our guide, presuming it to be at least as reliable as any other.

It appears that near seventy dentists enrolled their names on the Treasurer's list, which is quite encouraging, in the present state of our affairs. Still, we are not quite prepared to indorse the statement of Dr. J. D. White, that "Never before, perhaps, did so large a body of men assemble together and discuss the various topics of their profession or pursuit with so much good feeling toward each other;" and if Dr. W. had attended all the meetings of the Convention, he would have seen larger bodies of men manifesting quite as much good feeling as was possible at New Haven.

The old question relative to the admission of members was revived. It is, indeed, a ghost that refuses to be laid, but it is now, we hope, quieted by the adoption of a constitution. The proceeding, as reported, was the appointment of a committee to "revise" the constitution; but, unless we are mistaken, there was no constitution to revise. At its first organization, the Convention adopted a constitution, which was afterward totally set aside; and we have no recollection of another being adopted in its place.

The first subject for general discussion was PROFESSIONAL ETIQUETTE; and after some pointed remarks in reference to its importance by Dr. Whitney, all of which will be fully indorsed by the profession, Dr. Atkinson set forth the "golden rule" as the only safe and infallible guide in regard to the subject. This rule properly interpreted, will answer for all the departments of this life, certainly, and is not likely to go into disrepute, even in the life to come. But from some of the succeeding remarks on this rule in endeavors to prove its insufficiency, we are almost forced to conclude that there is a misconception, or difference of opinion as to what the rule is. One speaker, world renowned at that, thinks the golden rule would "not in all cases answer in this matter of etiquette," and gives in illustration, a case of neuralgia, in which a physician and dentist are separately consulted, and give diverse opinions, the conduct of the latter being wrong, because he did not ascertain that the other had been consulted, and advise with him. Now if there is any law on earth that would require the course suggested, it is the "golden rule."

Another, Dr. J., regarded the golden rule as "one of great beauty, but thought there were circumstances in which it would not apply." He, accordingly, suggests, as a substitute, uniform "*kindness*," and strict "*justice*."

We take it for granted that Dr. A., in referring to the "golden rule" intended that saying of our Savior recorded in Matt. vii. 12; and, if this was the understanding of all, how is it possible that Drs. H. and J. failed to see its nice adaptation to the cases supposed by them in endeavoring to prove its inapplicability? Does the golden rule not require "kindness" and "justice?" The Savior gives it as a summary of moral duty. "This is the law and the prophets." Does the *law*—God's moral code—not enjoin *kindness and justice?* Would the "law and the prophets" fail to teach the physician and dentist how to act in the case supposed by Dr. H.? Just here we referred to the nucleus of our library—the first volume we ever owned—and there we find the "golden rule" paraphrased as follows:—

> "Be you to others *kind* and *true*,
> As you'd have others be to you;
> And neither do nor say to men
> Whate'er you would not take again."

The book is called the "New England Primer." Its authorship is unknown to us; but when the martyr, John Rogers, the hero of the "nine small children, and one at the breast," whose fire and fagots horrified its pages, said

> "I leave you here a little book
> For you to look upon."

In our childish innocence we regarded our "Primer" as the identical "little book," and have, accordingly, kept it, that we might "look upon" it, ever since. And though

> "It was a childish ignorance,
> Yet now 'tis little joy
> To know ——— "

We were mistaken; but we never knew any one who followed the text or the paraphrase, who was chargeable with violation of etiquette. The man who obeys this rule, in his intercourse with society, is a perfect gentleman; and when a better guide, in etiquette, is devised, we may look for the discovery of a better illuminator than the sun. The many abortions in the shape of proposed codes of ethics in other professions, even more than in our own, when all that is required is simply to be *decent*, make us think

> "How well it is the sun and moon
> Are placed so very high
> That no presuming hand can reach
> To pluck them from the sky!
> Were it not so, I do believe
> That some reforming ass
> Would soon attempt to take them down,
> And light the world with gas."

The President read a paper on the "Causes which retard Dental Progress." The paper is interesting, progressive and practical, as well as historical and eulogistic. But it rather overdoes the matter when, in speaking of Fulton, it states:—"That which he saw only within the small compass of his brain, we can now see in the forms of steamships, steam cars, steam mills, steam factories, of such gigantic proportions as to fill the mind with awe, wonder, and surprise."

Now the truth is that some of the things here enumerated, for example, the *steam cars*, Fulton didn't see at all; and, though he may have seen steam mills and factories "in the small compass of his brain," he saw them as well, in full operation, on *terra firma.* And we feel that our favorite sciences are scarcely complimented by the President, when he speaks of chemistry, mineralogy and metallurgy as "minor auxiliaries" to dental science. But the President's paper filled a niche; and we are glad he prepared it.

Two papers were read by Dr. Atkinson, which would have been as appropriate at any other meeting as at a dental convention; but we like to read them, for reasons given heretofore. Dr. Burras read a paper on "Mastication, and Articulation of Artificial Dentures," which is a good deal anatomical, and a little practical, and will well repay perusal. Besides these, there was something read, which the *Cosmos* reporter, with a stretch of that charity which "believeth all things—hopeth all things," calls "an amusing poetical paper," of which we can only say that the fact of its being read at a meeting of the American Dental Convention, in classic New Haven, is profoundly humiliating.

The remarks of Drs. White, Atkinson, Wetherbee, Priest, and others, in regard to the importance of preserving the deciduous teeth are well worthy of attention; and we hope the younger members of our profession will cultivate that firmness recommended and practiced by Dr. Weatherbee. Parents are often unreasonable in their demands; but the dentist should never be dictated to. When our patrons will not rely on our judgment, after we have done all we can to enlighten them, we let them go. No man can afford to have such patrons. The suggestion of Dr. Priest, in regard to the education of parents, is well worthy of consideration. We can speak from experience in regard to its importance. The circulation of a few hundred copies of a popular essay among our patrons, has worked wonders. Those who have studied it take good care of their teeth, and, of course, have them filled in due time. And when they call on the dentist they know what they want, and are much better satisfied with good work than those less informed.

Dr. Woolworth has noticed that "where deciduous teeth were extracted early, the permanent ones would also come through too soon," which is just the reverse of our own observation; therefore we would be glad to hear from others on the subject. An extended series of observations would, no doubt, elicit the truth.

The subject of bleaching teeth was considered on the afternoon of the second day.

Dr. J. D. White thought the discoloration which takes place in teeth which have lost their pulps is not from the hæmatine, or coloring principle of the blood, but from the absorption of the fluids of the mouth. He opposes the use of the hypochlorite of soda, because it destroys the normal structure of the teeth. The hypochlorites do act with considerable energy on the dental tissues; but it is hard to get an effective bleaching agent that does not.

Dr. Barker differs with Dr. W., in regard to the discoloration of pulpless teeth, and gives a case of successful bleaching by the use of prepared chalk and hypochlorite of lime. It is not probable the chalk had any thing to do with the process; and the hypochlorite of lime has the same effect, and is, therefore, as objectionable as Labaraque's solution.

Dr. Atkinson tells us that "color is not an entity, but an arrested fractional part of light," which looks like reaching into the subject, but the next time he will modify his definition and call it a *rejected* fraction. His method of bleaching is to remove all the decayed material, and fill the cavity with "*undeliquesced* chloride of zinc, crowded into the cavity of the tooth." And at this point the bleaching properties of this agent were questioned, or rather doubted; and, if the report here is faithful, none of the speakers seem to have very clear ideas on the subject. While maintaining, (correctly enough) that chlorine is the bleaching agent, Dr. A. takes the position that undeliquesced chloride of zinc absorbs water from the mouth, and, by becoming liquid, is decomposed, its chlorine being thus liberated to effect the desired bleaching. From hearing his views heretofore, we infer that this statement does not do him justice; but it is only with the report, as such, that we have to do. The succeeding speaker, however, seems to be equally unhappy in his explanation. He tells us that it is " doubtful whether this action (bleaching) is due to the chlorine itself," which, perhaps, nobody claims. That is, all will admit that the oxygen, liberated when the chlorine takes hydrogen from water, has affinities sufficiently strong to decompose most organic coloring compounds which contain carbon and hydrogen. But its affinities, even in its nascent state, are not more energetic than chlorine. He tells us " moreover, the active agent in both the chlorides of lime and soda is not chlorine as such, but hypochlorous acid, which acid is set free from combination by any other acid, even the carbonic from the atmosphere." Now, if the professor had told that this acid "is very unstable, a slow decomposition taking place at common temperatures, by which chlorine is evolved"—that "this change is promoted by light, and is affected instantly by exposure for a few moments to the rays of the sun"—that it is also decomposed " by agitation with angular bodies" the decomposition being so rapid sometimes, as to cause effervescence by the escape of chlorine, we would have been hard to persuade that the active bleaching agent in the hypochlorites of lime and soda is not *chlorine, as such.* The great secret of the bleaching power of hypochlorous acid is its ready decomposition, by which the chlorine and oxygen are furnished so as to act in their nascent state. But it may be dangerous to differ with a man so learned that *he has no opinions.* But the professor's explanation of the action of chloride of zinc in whitening teeth, is peculiar. " His impression was that it acted physically in presenting a white ground in the tooth, and perhaps assisted to soften and remove animal coloring matters."

Now when the chloride deliquesces, which it soon does, it ceases to present a white ground; and it would be interesting to know just how it could *soften* and *remove* animal coloring matter. But this will suffice.

Under the head of "Filling Teeth and Roots," Dr. White claims that a great deal is gained by filling the roots of teeth, which is certainly true in many cases. He tells us "that metal well packed will shut out the gases and fluids which decompose and injure the structure of the teeth, also closing a cavity that may become filled with pus;" but he gives us a curious idea of "metal well packed" when he tells us that he "regarded it best so to fill fangs that it may be removed in case of trouble." It strikes us that a metal stuck into a canal so loosely that it can be drawn out at pleasure, is not so *well packed* that it will shut out gases and fluids—hardly exclude "that old chap, dampness," we fear. But the doctor subsequently "explained his manner of rolling a piece of gold, so as to be easily introduced and removed from the fangs of teeth," which is all very nice if any thing was gained by such *stuffing.* It is true that with gold lying loosely in the canal, it will not hold as great a quantity of "gases and fluids," but we can not see what else is gained.

Dr. White does not fill immediately after removing the pulp, while Dr. Weatherbee does. The latter claims extraordinary success—"in no case, during the past ten years, had he seen a single unfavorable case"—which laid a good foundation for the suggestion of Dr. Perine, "that we have no means of knowing how many unfavorable cases fall into the hands of other practitioners." We never admire a practice that is too successful. It argues a lack of opportunity or disposition to observe results.

The discussion on "plastic materials" for filling teeth appears to have elicited nothing of practical importance, unless it be that a tooth *sound enough* may possibly be improved by soaking it for three years in a solution of chloride of zinc. And it is not strange that the man who claims to be "the first in this country to use chloride of zinc," should speak of its removing the animal matter from a tooth?

The remainder of the report is interesting and practical, but presents nothing that calls for special notice here.

